# Adherent Placenta Retained Three Weeks without Putrefaction

**Published:** 1873-05-01

**Authors:** David Prince

**Affiliations:** Jacksonville, Ill.


### ADHERENT PLACENTA RETAINED THREE WEEKS WITHOUT PUTREFACTION.

SPECIMEN EXHIBITED BEFORE THE MORGAN COUNTY MEDICAL SOCIETY, WITH REMARKS.

BY DAVID PRINCE, M.D., JACKSONVILLE, ILL.

The report of Dr. H. Wardner, of Cairo, in the number of THE EXAMINER for March 15, induces me to report the following case, in which the placenta maintained its attachment and its vitality for three weeks from the time of the expulsion of a five months' fœtus.

Mrs. M., of hysterical temperament, but corpulent habit, mother of several children, patient of Dr. H. Jones, became unusually large after conception, and continued to have sanguineous discharges with the return of each monthly period. Doubt was thus thrown upon the nature of the case until one morning, going across the yard for what the patient supposed a call for urinary evacuation, a sudden pain seized her, and a fœtus was expelled. Grasping this through her clothing she walked back into the house and placed herself upon a lounge.

Owing to the sickness of Dr. Jones, the care of the patient was imposed upon me. About half an hour after this catastrophe, the fœtus was found attached by the umbilical cord, but a very slight force detached the cord so that the best guide to the placenta was unfortunately lost.

The uterus was explored with a placenta forceps, and none being found it was supposed that it must have been expelled and thrown away unobserved. The patient rapidly recovered and walked about the house, supposing that all trouble had passed by.

Just three weeks from the time of the abortion this lady was overtaken by a profuse hæmorrhage which subsided spontaneously. The horizontal posture was carefully maintained, and ergot was given. In a few hours a placenta, with its membrane complete, only showing a rent, was expelled in connection with coagula.

There was no more hæmorrhage; and though the skin was very pale from loss of blood, the recovery was speedy and complete. The placenta was altogether free from putridity; was compact, and destitute of apparent disease. One margin was dark with the presence of coagula, indicating the probable fountain from which the blood had gushed.

The location of the placenta upon the inner uterine surface cannot be certainly made out; but it is probable that it was so far toward the neck that the margin was detached by the expansion of the uterus with each monthly congestion.

It is possible that, in connection with the monthly hæmorrhages, there may have been such a change of the uterine surface of the placenta as to destroy the line of clearage.

If the stem of a leaf is wounded just at the line at which it will subsequently separate from the branch, it may readily be conceived that the healing process may obliterate this line of separation, so that the leaf, instead of falling off at the proper time, will maintain its vitality for a long period, and perhaps finally shrivel up without separation. The change which occurs in the leaf-stem may occur in the embryo-stem, permitting its base, the placenta, to maintain its vitality for several weeks after the falling of the embryo-leaf.

Dr. Hodges, in his folio work on Obstetrics, says that there is abundant proof that the placenta has adhered and maintained its vitality for weeks and even for months after the expulsion of the fœtus.

One of the practical inferences from this case is that it is not imperatively necessary to remove an adherent placenta by forcible means. Like the base of a polypus, it may be left to shrivel and separate by a natural process, which saves the uterus from the infliction of laceration and contusions.

---

FRECKLES, it is said, are removed by the application, twice a day, of powdered nitre moistened with water.

TWENTY drops of water have the same weight as 38 of laudanum, 58 of the ordinary tinctures and 100 of ether.

